# Supplementary material for: Differentiating Peromyscus leucopus bone marrow-derived macrophages for characterization of responses to Borrelia burgdorferi and lipopolysaccharide
Source: Infect Immun. 2025 May 27;93(7):e00581-24. doi: 10.1128/iai.00581-24 (PMC12234436; doi:10.1128/iai.00581-24)
Supplement: Supplemental material — Legends for all Supplemental data. [file iai.00581-24-s0010.docx]

**SUPPLEMENTAL MATERIAL LEGENDS**

**Supplemental File 1**: Plasmid map of JSB_Ec11 *P. leucopus* M-CSF producing construct

**Supplemental File 2**: *P. leucopus* BMDM differentially expressed gene expression analysis in response to *B. burgdorferi*

**Supplemental File 3**: C57BL/6J BMDM differentially expressed gene expression analysis in response to *B. burgdorferi*

**Supplemental File 4**: *P. leucopus* BMDM differentially expressed gene expression analysis in response to LPS

**Supplemental File 5**: C57BL/6J BMDM differentially expressed gene expression analysis in response to LPS

**Supplemental File 6**: Transcript Per Million data for C57BL/6J & *P. leucopus* BMDMs

**Supplemental File 7**: BMDM differentially expressed gene analysis, restricted to genes present in *P. leucopus* and C57BL/6J datasets, in response to *B. burgdorferi*

**Supplemental File 8**: QIAGEN Ingenuity Pathway Analysis for *B. burgdorferi* and LPS stimulated cells

**Supplemental File 9**: BMDM differentially expressed gene analysis, restricted to genes present in *P. leucopus* and C57BL/6J datasets, in response to LPS
